# Sustaining the workforce: job retention and workforce challenges among community pharmacists in Northern Ireland

**DOI:** 10.1080/20523211.2026.2677776

**Published:** 2026-06-01

**Authors:** Kingston Rajiah, Eoghan Roe O Neill

**Affiliations:** School of Pharmacy and Pharmaceutical Sciences, Ulster University, Coleraine, UK

**Keywords:** Community pharmacy, pharmacist workforce, job retention, workforce sustainability, career progression, Northern Ireland

## Abstract

**Background:**

Community pharmacists are vital to healthcare delivery in Northern Ireland, providing accessible patient care and public health services. However, the sector faces increasing challenges relating to job satisfaction, retention, and workforce stability. Existing evidence highlights systemic pressures, funding constraints, and limited career progression, but little is known about the lived experiences of pharmacists in Northern Ireland.

**Methods:**

This qualitative study used a descriptive design and thematic analysis to explore community pharmacists’ perspectives on workforce challenges and retention. Semi-structured interviews were conducted with 15 pharmacists from across all six counties of Northern Ireland between January and May 2025. Participants were purposively sampled to ensure diversity in age, experience, and pharmacy type. Interviews were audio-recorded, transcribed verbatim, and analysed inductively following Braun and Clarke’s six-step framework.

**Results:**

Six overarching themes were identified: (1) Professional fulfilment and identity, (2) Workplace culture and relationships, (3) Systemic pressures and workload burden, (4) Financial strain and limited career progression, (5) Workforce dynamics and generational shifts, and (6) Role development and professional support needs. Pharmacists derived satisfaction from patient rapport and role variety but reported these motivators were increasingly undermined by medicine shortages, workload intensity, unsustainable funding, and lack of structured career pathways. Generational changes and the appeal of locum work added further complexity, while enthusiasm for role expansion, particularly prescribing, was constrained by liability concerns and insufficient recognition.

**Conclusion:**

Community pharmacists in Northern Ireland remain motivated by professional fulfilment and patient-centred care, but systemic, financial, and structural barriers threaten retention. Sustainable funding, digital innovation, clear career progression, and enhanced professional recognition are urgently needed to support workforce stability and ensure the future of community pharmacy services.

## Background

Community pharmacy forms a cornerstone of healthcare delivery in Northern Ireland, providing highly accessible services to patients and acting as the first point of contact for medicines advice, minor ailments management, and public health interventions (Betts, 2016). Pharmacists are increasingly recognised not only for their dispensing role but also for their expanding clinical responsibilities, including vaccination delivery, independent prescribing, and minor illness management (Department of Health, 2015). As health systems face rising demand and workforce shortages, community pharmacists are expected to play an even greater role in bridging gaps in primary care. However, these growing responsibilities are accompanied by persistent workforce challenges, particularly around job satisfaction, retention, and recruitment.

Concerns about the sustainability of the pharmacy workforce are not unique to Northern Ireland. In the United Kingdom, the vacancy rate for community pharmacists has increased (Clews, 2023). In England, the National Health Service (NHS) England’s Community Pharmacy Workforce Survey 2022 reported a 13% reduction in full-time equivalent community pharmacists compared with the previous year, highlighting increasing difficulty in sustaining adequate staffing levels (Connelly, 2023). In the Republic of Ireland, research has shown that retention is negatively influenced by commercial pressures and personal accountability for professional activities (Lynch & O’Leary, 2023). International reviews have similar findings, with burnout, workload pressures, and limited career progression cited as key factors driving pharmacists away from community practice (Barakat & Sallam, 2025; Terry et al., 2021).

The Northern Ireland Department of Health’s Pharmacy Workforce Review (2020) identified significant challenges in community pharmacy, with two-thirds of pharmacists reporting no opportunities for career progression (Department of Health, 2020). The review also warned of an impending shortfall of 500–800 pharmacists by 2024, despite a 30% increase in the register between 2009 and 2020. Nearly 400 pharmacists have already left community practice since 2016, seeking alternative opportunities in hospital, general practice, or relocating to Great Britain and the Republic of Ireland, where salaries and conditions are often more favourable. Factors contributing to this attrition include unsociable working hours, high stress levels, low pay relative to responsibility, inadequate government funding, and limited career progression opportunities. These challenges are compounded by systemic pressures such as medicine shortages, rising regulatory and Continuous Professional Development (CPD) demands, and the absence of Electronic Transfer of Prescriptions (ETP), all of which intensify workload and erode job satisfaction.

The Health and Social Care Workforce Strategy 2026 stresses the importance of sustainable career pathways, workforce wellbeing, and role innovation across the health system (Health and Social Care, 2018). Within this context, community pharmacy is experiencing significant cultural and generational change. Younger pharmacists increasingly prioritise work-life balance, flexibility, and wellbeing, whereas older pharmacists often entered the profession with expectations of long-term commitment to a single sector. While locum work offers freedom and adaptability, it can reduce continuity of patient care and undermine long-term workforce stability. These shifting dynamics underscore the urgent need to examine the factors influencing retention and to develop strategies that can sustain a motivated and resilient community pharmacy workforce in Northern Ireland.

Job retention is not only a matter of individual career choices but also of system-level sustainability. High turnover undermines continuity of care, increases costs associated with recruitment and training, and risks destabilising pharmacy services at a time when demand is at its highest. Retention depends on more than pay alone; it is shaped by a combination of professional fulfilment, workplace culture, systemic workload pressures, financial viability, and opportunities for role development. Understanding how these factors interact is essential for policymakers, professional bodies, and employers seeking to implement targeted interventions.

This study, therefore, seeks to explore community pharmacists’ perspectives on job satisfaction, retention, and workforce challenges in Northern Ireland. Using a qualitative descriptive design and thematic analysis, the research captures pharmacists’ lived experiences in their own words, highlighting both the barriers to retention and the enablers that support workforce sustainability. In doing so, the study provides timely insights to inform workforce planning and the development of policies aimed at retaining a resilient and motivated pharmacy workforce.

## Methods

### Study design

This study adopted a qualitative descriptive design, guided by an inductive thematic analysis approach as outlined by Braun and Clarke (Braun & Clarke, 2006). This approach was considered appropriate as it provides a straightforward means of capturing and presenting participants’ views in their own words, particularly in professional areas where little prior research exists. Thematic analysis offered a flexible yet rigorous method for identifying, analysing, and reporting patterns within the data, allowing the researchers to develop a rich understanding of pharmacists’ operational realities without imposing pre-existing theoretical frameworks.

### Research setting and context

The research took place among community pharmacists working across Northern Ireland, encompassing all six counties. The healthcare system in Northern Ireland integrates community pharmacies into the wider public health service, operating within a centralised prescription model and a mixed economy of independent and corporate-owned pharmacies. Community pharmacies are recognised as highly accessible points of care and increasingly take on additional clinical responsibilities, making them a key focus for workforce research.

### Participant recruitment

A purposive sampling strategy was used to ensure the inclusion of pharmacists with diverse backgrounds and experiences. Efforts were made to include participants across a range of ages, workplace settings (independent and chain pharmacies), geographical locations (all six counties), and varying years of experience. To be eligible, participants had to be registered pharmacists working in community pharmacy in Northern Ireland with at least one year of post-registration experience. Recruitment was carried out through professional pharmacy networks, email invitations distributed via representative bodies, and direct contact with pharmacy leads. No financial incentives were offered for participation. In total, 15 pharmacists took part in the study. Thematic saturation, defined as the point at which no new codes were identified during analysis, was reached by the thirteenth interview. Two further interviews were conducted to confirm the stability of the themes.

### Data collection

Data were gathered through semi-structured, in-depth interviews conducted online using Zoom or Microsoft Teams, depending on participant preference. This approach offered flexibility and accessibility, reducing barriers for participants working under high workload conditions. The interview guide was developed following a review of relevant literature and was structured around broad areas, including career trajectory, job satisfaction, workforce challenges, retention factors, and views on future workforce planning. The guide was piloted with two pharmacists, which resulted in refinements to improve clarity and flow, such as simplifying technical terms and reordering questions to encourage a more natural conversational style. Field notes were taken immediately after each interview to capture contextual details, including tone, emphasis, and nonverbal cues (where visible). Interviews lasted between 30 and 45 min, were audio-recorded with the participant’s informed consent, and transcribed verbatim. All transcripts were anonymised during transcription to protect participant confidentiality. Interviews were conducted between January and May 2025.

### Data analysis

Thematic analysis was undertaken following Braun and Clarke’s six-phase framework, adopting an inductive and semantic approach (Braun & Clarke, 2006). This meant that coding was driven by the data itself rather than a pre-existing theory, and that codes reflected the explicit meanings expressed by participants. Analysis began with familiarisation, where all transcripts were read multiple times by the research team, accompanied by reflective note-taking to record early impressions and potential patterns. Initial coding was conducted independently by the lead researcher, working line-by-line through the transcripts to identify meaningful segments of data. Each segment was assigned a short descriptive label, forming the basis of a shared codebook. This codebook was updated iteratively through weekly research team meetings, where codes were discussed, refined, merged, or split as necessary. Codes that did not initially fit with emerging patterns were set aside for later review.

**Figure 1.**
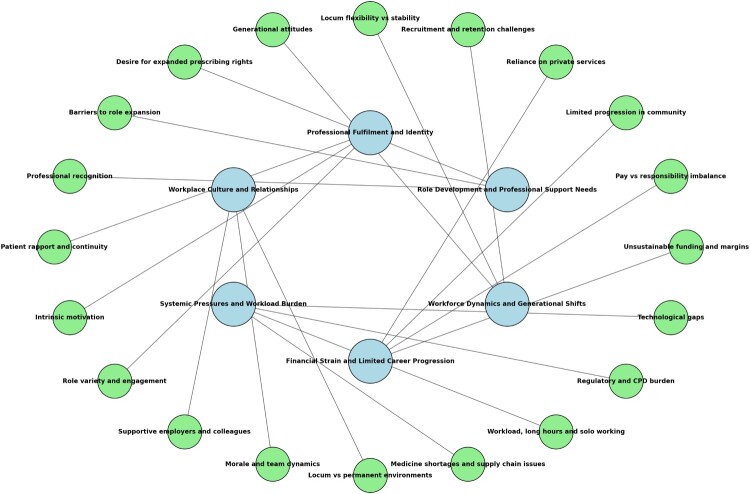
Visual mapping of themes and sub-themes.

Once coding was complete, related codes were grouped into candidate themes through an iterative clustering process. Visual mapping techniques were used to explore relationships between codes, and related concepts were brought together into broader categories (see Figure 1). For example, codes such as ‘lack of continuity with patients,’ ‘desire for prescribing rights,’ and ‘fear of liability’ were clustered under the theme Role Development and Professional Support Needs. Themes were then reviewed for internal consistency and distinctiveness, with subthemes identified to highlight different layers of meaning. Outlier or negative cases were also examined to refine theme definitions and ensure the analysis reflected both common and divergent perspectives. Each theme was then defined and named in a way that captured its core meaning and scope. Saturation was confirmed at interview thirteen, with interviews fourteen and fifteen used to verify thematic stability. In the write-up, interpretation of the data was combined with direct participant quotations, chosen to illustrate key points and provide insight into pharmacists’ lived experiences. All coding was done manually without the use of qualitative data analysis software. Any differences in coding were resolved through consensus, and peer debriefing with the wider research team occurred fortnightly to challenge interpretations and maintain reflexivity.

### Trustworthiness and rigour

The study adhered to Lincoln and Guba’s four criteria for trustworthiness: credibility, transferability, dependability, and confirmability (Forero et al., 2018). Credibility was supported through investigator triangulation, regular peer debriefing, and the use of direct participant quotations to ground findings in the data. Transferability was enhanced by providing detailed descriptions of the research setting, participant demographics, and context. Dependability was ensured by maintaining an audit trail of coding decisions, iterations of the codebook, and theme refinement processes. Confirmability was supported through reflexive journaling, the use of negative case analysis, and secure retention of anonymised raw data for verification purposes. Member checking was considered but not undertaken, as the ethics committee advised it could compromise participant confidentiality within a small professional community and place additional time burdens on participants. However, the research team engaged in repeated discussions to ensure that the final themes accurately reflected participants’ voices.

### Reflexivity

The chief researcher is a pharmacy professional with experience in public health, pharmacy practice, and qualitative research. While this professional background provided valuable contextual insight, it also carried the risk of bias in interpreting participants’ accounts. To address this, the lead researcher maintained a reflexive journal to record assumptions, decisions, and reflections after each interview. Another member of the research team brought expertise in community pharmacy and qualitative methodology, which helped challenge interpretations and reduce bias. Team discussions actively considered how personal and professional experiences might influence the analysis, and a conscious effort was made to ensure that participants’ voices were prioritised throughout the reporting of findings.

## Results

A total of 15 community pharmacists participated in the study, representing a balanced representation across age groups, genders, levels of experience, job roles, and geographical distribution. Participants ranged in age from 25 to over 45 years, with equal representation across the three age brackets. Just over half of the participants were female, and professional experience ranged from 1 to more than 20 years. The sample included both locum pharmacists and pharmacist managers, working across urban and rural locations, with at least two participants recruited from each of the six counties in Northern Ireland. These characteristics ensured diversity in perspectives and enhanced the transferability of findings. A detailed breakdown of participant demographics is presented in Table 1.

**Table 1.** Demographics of participants.

Demographic Characteristics	Frequency (*n* = 15)	Proportion %
Age Range in years		
25–30	5	33.3
31–40	5	33.3
41 and above	5	33.3
Gender		
Female	8	53.3
Male	7	46.7
Experience in years		
1–5	5	33.3
6–10	4	26.7
11–20	6	40
Job description		
Locum Pharmacist	6	40
Pharmacist	5	33.3
Pharmacist Manager	4	26.7
Pharmacy location		
Urban	7	46.7
Rural	8	53.3
County		
Antrim	3	20
Armagh	2	13.3
Down	2	13.3
Fermanagh	2	13.3
Londonderry	3	20
Tyrone	3	20

### Theme 1: professional fulfilment and identity

Participants expressed strong professional fulfilment, often tied to their role as accessible healthcare providers within the community. Many described a sense of identity shaped by patient contact, continuity of care, and the ability to make a difference in people’s lives. Despite the pressures of the role, participants frequently highlighted a deep-rooted motivation to remain in the profession. For some, diversifying their practice into new services helped sustain this engagement.

#### Subtheme 1.1: patient rapport and continuity

- ‘The rapport you build with people is what keeps me in this role.’ (P1)
- ‘I’ve seen families grow up over the years – that’s the most rewarding part.’ (P5)

#### Subtheme 1.2: intrinsic motivation

- ‘I still love the job – even with all the pressures, it’s rewarding to help patients.’ (P3)
- ‘Helping people and being that first point of contact is what motivates me.’ (P12)

#### Subtheme 1.3: role variety and engagement

- ‘Running clinics and prescribing makes the role more interesting – it stops you getting stuck in a rut.’ (P5)
- ‘I like the freedom to launch new services; it gives me a sense of ownership.’ (P3)
- ‘I enjoy the hustle and bustle of community – it suits me better than a quiet role.’ (P13)

### Theme 2: workplace culture and relationships

The workplace environment was seen as central to retention. Supportive colleagues and approachable employers created a positive culture, while poor morale or unsupportive management contributed to dissatisfaction. Locum pharmacists in particular valued the ability to choose where they worked, allowing them to avoid negative environments and prioritise more positive workplace cultures.

#### Subtheme 2.1: supportive employers and colleagues

- ‘A good boss makes all the difference – someone approachable and fair.’ (P2)
- ‘Being part of a family business means you’re supported and everyone pulls together.’ (P11)

#### Subtheme 2.2: morale and team dynamics

- ‘Keeping the staff motivated is one of the hardest things.’ (P1)
- ‘A small gesture like bringing in food goes a long way to lift morale.’ (P4)

#### Subtheme 2.3: locum vs permanent environments

- ‘If I don’t like a pharmacy, I just don’t book there again.’ (P2)
- ‘I’ve locumed in over 60 pharmacies – the environments vary massively.’ (P14)

### Theme 3: systemic pressures and workload burden

Pharmacists consistently reported feeling weighed down by systemic and structural challenges. Workload intensity, medicine shortages, regulatory demands, and outdated systems were seen as major obstacles to job satisfaction. These pressures often overshadowed the positive aspects of the role, creating a sense that the profession is becoming increasingly unsustainable without change.

#### Subtheme 3.1: medicine shortages and supply chain issues

- ‘It’s an endless battle sourcing medicines – it takes me away from patient care.’ (P1)
- ‘I can spend hours each day chasing stock – it’s soul-destroying.’ (P11)

#### Subtheme 3.2: workload, long hours, and solo working

- ‘Some days it’s overwhelming being the only pharmacist on duty.’ (P4)
- ‘The workload has increased massively since Covid.’ (P15)

#### Subtheme 3.3: regulatory and CPD burden

- ‘CPD hours keep going up – the burden is huge compared to the support we get.’ (P1)
- ‘So much of our time is swallowed by paperwork rather than patients.’ (P8)

#### Subtheme 3.4: technological gaps

- ‘EPrescribing would transform the workload – it’s frustrating we don’t have it yet.’ (P4)
- ‘We’re still stuck with paper processes, which slows everything down.’ (P7)

### Theme 4: financial strain and limited career progression

Financial concerns were a dominant theme, with participants highlighting that community pharmacy business models are increasingly under pressure. Many felt their level of responsibility was not matched by remuneration, and compared unfavourably with hospital or GP settings, which offered clearer pathways for career advancement. As a result, community pharmacy was sometimes described as offering limited prospects unless supplemented by private services.

#### Subtheme 4.1: unsustainable funding and margins

- ‘Margins on dispensing are so poor, you can’t survive on NHS work alone.’ (P3)
- ‘The clawback and funding cuts are killing community pharmacy.’ (P7)

#### Subtheme 4.2: pay vs responsibility imbalance

- ‘We carry the can for everything, but the pay doesn’t reflect that responsibility.’ (P1)
- ‘Pharmacists in Northern Ireland are the lowest paid in the British Isles.’ (P10)

#### Subtheme 4.3: limited progression in community

- ‘In hospital, there’s a clear career ladder – in community, there isn’t.’ (P1)
- ‘Community feels like a dead end if you want to progress clinically.’ (P9)

#### Subtheme 4.4: reliance on private services

- ‘Private services are the only way to keep the business afloat.’ (P3)
- ‘New services help, but they don’t cover the shortfall in NHS funding.’ (P12)

### Theme 5: workforce dynamics and generational shifts

Participants noted that workforce patterns and expectations are shifting, both in recruitment and in career outlook. Participants reported increasing difficulty in attracting and retaining staff, while generational differences emerged in attitudes toward career commitment, work-life balance, and flexibility. Locum work was often seen as appealing for younger pharmacists, though it lacked stability and continuity of care.

#### Subtheme 5.1: recruitment and retention challenges

- ‘It’s harder to recruit now – many leave for hospital or GP roles.’ (P5)
- ‘Getting locum cover has become a nightmare.’ (P12)

#### Subtheme 5.2: locum flexibility vs stability

- ‘Locum work gives me flexibility, but it doesn’t give me continuity with patients.’ (P4)
- ‘I like the freedom of locum work early in my career – it lets you see what’s out there.’ (P9)

#### Subtheme 5.3: generational attitudes

- ‘Younger pharmacists aren’t as career-focused – they want balance and less responsibility.’ (P1)
- ‘The new generation just don’t want the same pressures we accepted.’ (P15)

### Theme 6: role development and professional support needs

Participants highlighted a strong appetite for expanding their clinical roles, particularly in prescribing. Many felt their skills were underused, but enthusiasm was tempered by concerns over liability, bureaucracy, and the lack of systemic support. Participants also felt their profession is undervalued by government and policymakers, and that greater recognition is essential for pharmacy to progress.

#### Subtheme 6.1: desire for expanded prescribing rights

- ‘Pharmacists should be prescribing more – it’s a wasted opportunity.’ (P4)
- ‘We’re doing half of the doctor’s job already – why not let us prescribe properly?’ (P6)

#### Subtheme 6.2: barriers to role expansion

- ‘I’d love to prescribe, but without union support the liability is too risky.’ (P4)
- ‘These new services are overly bureaucratic – they don’t make real use of our skills.’ (P14)

#### Subtheme 6.3: professional recognition

- ‘Until government recognises our contribution, pharmacy won’t move forward.’ (P1)
- ‘We’re undervalued – other professions get paid for their time, but we don’t.’ (P10)

## Discussion

This study explored community pharmacists’ perspectives on job satisfaction, retention, and workforce challenges in Northern Ireland. A consistent finding in this study was that professional fulfilment remains central to community pharmacists’ decisions to remain in the profession. Pharmacists described their professional identity as being shaped by meaningful patient interactions, continuity of care, and the ability to make a difference within their communities. Long term relationships and patient trust were key sources of job satisfaction, supporting previous evidence that continuity of care and strong patient-pharmacist relationships enhance satisfaction and protect against burnout (Alshahrani et al., 2025; Katsogiannis et al., 2024). Intrinsic motivation also played an important role, with pharmacists valuing their position as a first point of contact in healthcare. Similar findings have been reported elsewhere, where professional identity has been identified as a core motivator for retention (Asghar et al., 2024; Ooi et al., 2023). However, existing literature cautions that intrinsic rewards alone may be insufficient to sustain long-term retention if workload and financial pressures persist.

Variety and role diversification were also important contributors to fulfilment. Pharmacists involved in prescribing, clinical services, or private clinics reported greater engagement and reduced risk of stagnation. This aligns with studies showing that expanded clinical roles enhance professional value and satisfaction (Cherecheș et al., 2024; Lott et al., 2021). Nevertheless, this study highlights that the benefits of role diversification depend on adequate staffing, time, and financial support. Without these, extended services may become an added burden rather than a retention strategy.

Workplace culture emerged as a key influence on job satisfaction and retention. Supportive colleagues and approachable employers fostered positive environments, while poor morale and unsupportive management contributed to dissatisfaction. These findings reinforce evidence that collegial relationships and leadership are strong predictors of job satisfaction across healthcare professions (Iqbal et al., 2025; Radwan et al., 2022). Maintaining morale was particularly challenging in high-pressure settings, although small acts of recognition were seen to improve team cohesion. Similar observations have been reported, where supportive workplace cultures reduced burnout and improved retention (Bahadur & Macdonald, 2025; Kiriazopoulos et al., 2025). Locum pharmacists valued the flexibility to avoid negative environments, although this flexibility was less available to permanent staff. While locum work offers adaptability, it is less supportive of continuity of care (Kausar, n.d.; Sanderson, 2022), highlighting the need for positive cultures across all pharmacy settings.

Systemic and structural pressures were major barriers to retention. Workload intensity, medicine shortages, regulatory demands, and outdated systems often overshadowed the positive aspects of the role. Medicine shortages were especially time-consuming and demoralising, diverting pharmacists from patient care and affecting patient trust. Similar challenges have been reported across the UK and internationally (Schommer et al., 2022; Willis, 2025). Workload pressures increased further due to staff shortages and expanded responsibilities following the COVID pandemic, consistent with research linking workload to burnout and attrition (Connelly, 2024; O’Donnell et al., 2024). Regulatory and CPD requirements added to this burden, particularly when perceived as disconnected from frontline realities (Langran et al., 2022). The continued reliance on paper-based systems and the absence of Electronic Transfer of Prescriptions in Northern Ireland further exacerbated inefficiencies, contrasting with evidence from England where electronic prescribing improved workflow (Goundrey-Smith, 2018).

Financial strain was another dominant concern. Pharmacists described unsustainable funding, shrinking margins, and clawbacks as major threats to business viability, reflecting wider UK evidence linking underfunding to service reductions and closures (Community Pharmacy England, 2025; Willis, 2024). Dissatisfaction was compounded by a perceived imbalance between responsibility and pay, with pharmacists feeling undervalued compared to hospital and GP colleagues (Bounthavong, 2024; Pharmacists’ Defence Association, 2021). Limited career progression further reduced retention, as community pharmacy was often viewed as lacking structured advancement pathways (Company Chemists’ Association, 2025; Robertson, 2024). Although private services offered some financial relief, they were not seen as a substitute for stable core funding (Castle-Clarke et al., 2017).

Finally, shifting workforce dynamics and generational attitudes were evident. Recruitment and retention challenges were widespread, with locum work appealing to younger pharmacists seeking flexibility and work-life balance (Locate A Locum, 2023; Trinh, 2024). While this may reduce burnout, it poses challenges for continuity and workforce stability. Pharmacists also expressed strong interest in role expansion, particularly prescribing, consistent with UK policy ambitions and evidence linking role development to satisfaction (Rosenthal et al., 2011). However, concerns about liability, bureaucracy, and lack of recognition remain barriers. Without adequate support and professional recognition, role expansion risks increasing strain rather than improving retention.

### Limitations of the study

This study provides important insights into community pharmacists’ experiences of job satisfaction, retention, and workforce challenges in Northern Ireland, but several limitations should be acknowledged. First, all data relied on self-reported accounts, which may be subject to recall bias or influenced by participants’ immediate work experiences at the time of the interview. Second, although the lead researcher maintained a reflexive journal and engaged in peer debriefing to reduce bias, the interpretation of data may still have been shaped by their professional background. Finally, interviews were conducted online, which, while convenient, may have limited the observation of non-verbal cues compared to in-person interviews. Despite these limitations, the study offers a rich and credible account of the lived realities of community pharmacists in Northern Ireland.

### Implications for policy and practice

The findings highlight urgent implications for workforce planning and policy in Northern Ireland. Professional fulfilment, workplace culture, and opportunities for role development remain critical enablers of retention, yet these strengths are increasingly undermined by systemic pressures, financial strain, and lack of recognition. The Northern Ireland Department of Health’s Pharmacy Workforce Review already identified the absence of career progression in community pharmacy (Department of Health, 2020). Our findings reinforce this concern and suggest that implementing structured pathways for advancement must be prioritised if workforce sustainability is to be achieved. Policymakers must prioritise sustainable funding models for community pharmacy to address financial instability and ensure that pharmacists are fairly remunerated relative to their responsibilities. Investment in digital infrastructure, such as electronic prescribing, is also essential to reduce workload inefficiencies and enable pharmacists to focus on patient-facing care.

At the organisational level, pharmacy employers should strengthen workplace cultures by supporting leadership development, recognising staff contributions, and fostering positive team dynamics. Structured career progression frameworks, similar to those in hospital pharmacy, are needed to retain pharmacists within the community sector and to provide younger generations with a compelling reason to build long-term careers in this setting. Finally, successful role expansion into areas such as prescribing must be matched with appropriate governance, liability protections, and professional recognition to ensure that new responsibilities enhance, rather than erode, job satisfaction.

### Recommendations for future research

Future research should build on these findings by adopting larger mixed-methods or longitudinal approaches to explore workforce dynamics in greater depth. Quantitative studies could measure the prevalence of the themes identified here, helping to assess their impact on retention across the wider pharmacist population. Longitudinal research would be valuable to track how job satisfaction and workforce intentions evolve over time, particularly as new policies such as independent prescribing and expanded clinical services are implemented. Comparative studies across different regions of the UK and Ireland would also help to contextualise Northern Ireland’s workforce challenges within broader trends. Finally, further exploration of generational differences in career expectations and motivations could provide important insights for tailoring retention strategies to different cohorts within the profession.

## Conclusions

Pharmacists reported strong professional fulfilment through patient rapport, continuity of care, and role variety. However, these motivators are increasingly undermined by systemic pressures, financial strain, and limited career development. Workplace culture was identified as a key influence on retention, with supportive teams and leadership fostering satisfaction, while poor morale and unsustainable workloads contributed to disengagement. Persistent medicine shortages, regulatory demands, and a lack of integrated digital infrastructure further reduced capacity for patient care. Financial instability, pay responsibility imbalance, and limited progression opportunities continue to drive pharmacists toward alternative roles. Generational shifts and the appeal of locum work add further complexity, while enthusiasm for role expansion, particularly prescribing, was tempered by concerns over liability and lack of recognition. Though professional fulfilment remains a strength of community pharmacy, it is insufficient to counter systemic and financial pressures. Sustainable funding, structured career pathways, digital innovation, and professional recognition are urgently required to support retention and ensure the long-term stability of the sector.

## Data Availability

The data that support the findings of this study are available from the corresponding author upon reasonable request.
